# Influence of orthodontic treatment with fixed appliances on enamel color: a systematic review

**DOI:** 10.1186/s12903-015-0014-x

**Published:** 2015-03-10

**Authors:** Qiushuo Chen, Xicong Zheng, Weiting Chen, Zhenyu Ni, Yu Zhou

**Affiliations:** Department of Orthodontics, Hospital of Stomatology, Wenzhou Medical University, No. 113 West College Road, Wenzhou, Zhejiang China

**Keywords:** Orthodontic treatment, Enamel color, Systematic review

## Abstract

**Background:**

The purposes of this systematic review were to identify and review the orthodontic literature with regard to enamel color alterations after orthodontic treatment with fixed appliances. The effects of clean-up procedures on the surface of enamel were also investigated.

**Methods:**

We searched the following electronic databases: Medline (1950 to 6 July 2014), EMBASE via OVID (1980 to 6 July 2014), Google Scholar, Web of Science (1950 to 6 July 2014), CENTRAL (The Cochrane Library, 2014, Issue 7). We also searched the reference lists of relevant articles. Quality assessment of the included articles was performed. Two authors were responsible for study selection, validity assessment, and data extraction.

**Results:**

Five studies met the inclusion criteria, including 3 randomized controlled trials and 2 prospective studies. Four trials were assessed as being unclear with regard to risk of bias. One was assessed as being at high risk of bias. The studies reviewed suggested that orthodontic treatment alters the original color of enamel, and both adhesive systems and resin-removal methods can contribute to this change.

**Conclusion:**

There is no strong evidence from this review that orthodontic treatment with fixed appliances alters the original color of enamel. Further well-designed and conducted randomized controlled trials are required, to facilitate comparisons of results.

## Background

Treatment with fixed orthodontic appliances has become dominant in orthodontic practice throughout the world. Epidemiological investigation [1] reveals that approximately 30% of the population has either a moderate or great need for orthodontic treatment. However, since the introduction of the acid-etch technique, and its utilization for the bonding of orthodontic brackets, returning the enamel surface to as near to its original state as possible with the minimum amount of enamel loss at the end of treatment has become a primary concern. Bonding, debonding, and clean-up procedures may result in enamel alterations such as enamel loss caused by etching [2], decalcification [3], and enamel scratches [4]. Besides structural defects, attention should also be paid to the adverse effects on the color and esthetics of enamel associated with the aforementioned changes [5]. However, despite extensive evidence of enamel loss [6-8], the incidence of enamel color changes induced by bonding and debonding procedures has not been thoroughly investigated.

Previous studies [4,9] have shown that enamel color variables are affected by enamel bonding and debonding procedures. Enamel color alterations may derive from post-debonding resin removal protocols [9], and the penetration of resin tags into the enamel structure at depths reaching 50 mm [4]. Resin impregnation into the enamel structure cannot be reversed by debonding and cleaning procedures [3], and enamel discoloration may occur by direct absorption of food colorants and products arising from corrosion of the orthodontic appliance [10]. The long-term presence of these resin residues in the enamel tags that extend over the middle third of the buccal surface may render the color stability of these materials critical for tooth color. Additionally, post-debonding protocols involving removal of adhesive residues with various rotary abrasive tools or hand-held instruments may increase the roughness of the enamel surface, which may lead to color alterations [11].

Although the results of these studies showed that some alterations in tooth color are inevitable, irrespective of whether these changes are visually recognizable, in vitro tests may not accurately reflect the clinical situation [12]. Natural tooth-color determination is affected by many factors in the oral cavity, such as the lighting conditions of the surrounding environment, the light scattered from adjacent periodontal and gingival tissues [13], and resting salivary flow rates, which influence tooth hydration [14], and consequently the reflective index of the underlying surface.

The determination of tooth color has always been problematic in dentistry. There are two common methods of analyzing tooth color in vivo: visual determination and instrumental measurement [15]. Visual determination by comparing teeth with shade guides is considered highly subjective, but remains the most frequently utilized method [16]. However, several factors such as external light conditions, experience, age, fatigue of the human eye, and the inherent limitations of contemporary shade guides can influence the consistency of visual color selection and specification [15-18]. The general demand for objective color matching in dentistry, coupled with rapid advances in optical electronic sensors and computer technology, has made instrumental measurement devices a supplementary adjunct to visual tooth color evaluation [19]. Nowadays, various commercial systems including tristimulus colorimeters, spectroradiometers, spectrophotometers, and digital color analyzers are used in clinical and research settings for the objective determination of color [20]. Therefore, there is a need for a systematic review to critically appraise and summarize the results of clinical trials, to assess the influence of orthodontic treatment with fixed appliances on enamel color.

## Methods

This systematic review was conducted according to the guidelines of the Preferred Reporting Items for Systematic Reviews and Meta-Analyses (PRISMA) statement and the Cochrane Handbook. A review protocol does not exist.

### Search methods for identification of studies

The databases Medline (1950 to 6 July 2014), EMBASE via OVID (1980 to 6 July 2014), Web of Science (1950 to 6 July 2014), Google Scholar, and CENTRAL (The Cochrane Library, 2014, Issue 7) were searched. For the identification of studies considered for inclusion in this review, we developed detailed search strategies for each database searched. These were based on the search strategy developed for MEDLINE (see Table 1), but revised appropriately for each database to take into account differences in controlled vocabulary and syntax rules. This search strategy was used in addition to the Cochrane Highly Sensitive Search Strategy (CHSSS) for identifying randomized trials in MEDLINE: sensitivity maximising version (2008 revision), as referenced in Chapter 6.4.11.1 and detailed in box 6.4.c of the Cochrane Handbook for Systematic Reviews of Interventions Version 5.1.0 (updated in March 2011) [21].

**Table 1:** **Search strategy**

1.		exp Enamel Colour/
2.		“enamel clour”.mp.
3.		enamel clour or enamel color.mp.
4.		“adhensive”.mp.
5.		(conventional acid-etching adj3 adhensive) or (conventional acid etching adj3 adhensive) or (self-etching primers adj3 adhensive) or (self etching primers adj3 adhensive).mp.
6.		“clean-up procedures” or “clean up procedures”.mp.
7.		Or/1-6
The above subject search was linked to the Cochrane Highly Sensitive Search Strategy (CHSSS) for identifying randomized trials in MEDLINE: sensitivity maximising version (2008 revision) as referenced in Chapter 6.4.11.1 and detailed in box 6.4.c of the Cochrane Handbook for Systematic Reviews of Interventions, Version 5.1.0 [updated March 2011]:		
1.		randomized controlled trial.pt
2.		controlled clinical trial.pt.
3.		randomized.ab
4.		placebo.ab.
5.		drug therapy.fs.
6.		randomly.ab.
7.		trial.ab.
8.		groups.ab.
9.		or/1-8
10.	exp animals/not humans.sh.	
11.	9 not 10	

We also manually searched relevant orthodontics journals (American Journal of Orthodontics and Dentofacial Orthopedics, European Journal of Orthodontics, Angle Orthodontist, Journal of Orthodontics, and World Journal of Orthodontics).We checked the bibliographies of included publications and relevant review articles for studies not identified by the above search strategies. We contacted the authors of studies designated for inclusion, to identify unpublished or ongoing trials.

### Selection of studies

For inclusion, studies were required to meet the following criteria:

#### Types of studies

We included randomized controlled trials (RCTs), and prospective controlled clinical studies.

#### Participants

We included participants with fixed orthodontic appliances. We excluded participants who had had previous active orthodontic treatment, or had a relevant medical history.

#### Interventions

Assessments of the influence of the type of orthodontic adhesive system on enamel color changes during bonding and after debonding. Effects of clean-up procedures on the enamel surface were also evaluated.

#### Outcome measures

Color changes before and after orthodontic treatment.

Studies other than RCTs or prospective controlled studies, studies that did not investigate enamel color changes or did not investigate fixed appliance interventions, and animal studies were excluded during the screening process. At least two authors independently assessed the list of titles and abstracts of potentially eligible studies. We obtained full-text versions of publications that fulfilled the inclusion criteria. Any disagreements were resolved by discussion or judged by a third reviewer.

### Data extraction and analysis

At least two authors assessed all included studies, to confirm eligibility, assess risk of bias, and extract data. The following data were extracted: Study design, participants, intervention, outcome measure. The risks of bias associated with each included study were independently assessed by two authors using the Cochrane risk of bias assessment tool. The potential sources of bias considered were sequence generation, allocation concealment, blinding of outcome and participants, completeness of outcome data, risk of selective outcome reporting, and risk of other potential sources of bias. Each of these domains included one or more specific entries in a “Risk of bias” table. Within each entry, what was reported in the study was described and a judgment relating to the risk of bias for that entry was assigned. Where the methodology of the study was clearly reported, a judgment of “Low risk” of bias or “High risk” of bias was made. Where trial methodology was unclear, the domain was assigned a determination of “Unclear risk” of bias, unless further information was available. After taking into account the additional information provided by the authors of the trials, we grouped studies into three categories (Table 2).

**Table 2:** **Categories of risk of bias**

**Risk of bias**	**Interpretation**	**Within a study**	**Across studies**
Low risk of bias	Plausible bias unlikely to seriously alter the results	Low risk of bias for all key domains	Most information is from studies at low risk of bias
Unclear risk of bias	Plausible bias that raises some doubt about the results	Unclear risk of bias for one or more key domains	Most information is from studies at low or unclear risk of bias
High risk of bias	Plausible bias that seriously weakens confidence in the results	High risk of bias for one or more key domains	The proportion of information from studies at high risk of bias is sufficient to affect the interpretation of results

We only conducted a meta-analysis of data from similar studies reporting the same outcome measures. We combined risk ratios for dichotomous data, and mean differences for continuous data, using random-effects models provided there were more than three studies in the meta-analysis. The I^2^ statistic was to be used to quantify heterogeneity, where I^2^ > 50% was considered substantial heterogeneity.

## Results

### Study selection and description of studies

**Figure 1:**
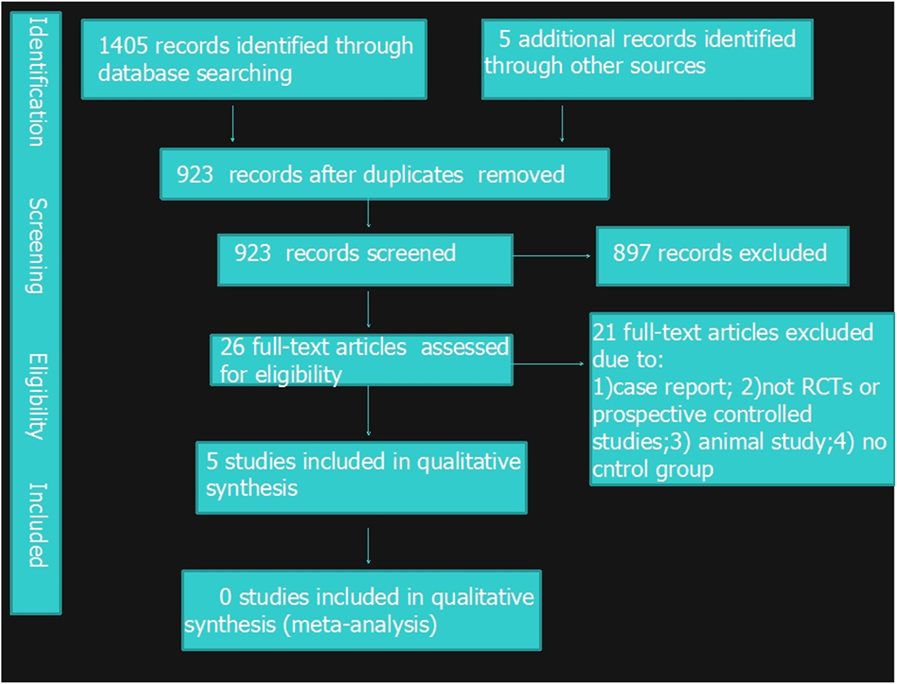
**Flow figure.**

The agreement between the two independent authors with regard to article screening was substantial (kappa = 0.922). A flow diagram depicting the results of the search queries is shown below (Figure 1). We initially identified a total of 1410 references and 26 reports of trials as eligible according to the defined inclusion criteria for this review. We obtained full text copies of these reports, and after further evaluation, the total number of trials which met our inclusion criteria was 5 [22-26]. The details of each of these studies are presented in Table 3.

**Table 3:** **Summarized data of the 5 included studies**

**Authors, year**	**Study design**	**Participants size, gender, age**	**Intervention, end point**	**Outcome measure**	**Outcome and authors conclusions**	**Imaging parameters**
Karamouzos 2010 [25]	Prospective split-mouth design	26 patients (13 girls, 13 boys) a mean age of 13 years 7 months (SD, 2.9 years)	Adhesives—chemically cured (System 11, Ormco, Glendora, CA, USA) and light-cured (Transbond XT, 3 M Unitek, Monrovia, CA, USA)	The resultant color differences (DE) between the interval groups were calculated	Chemically cured resin was associated with greater color changes than light-cured composite	The reflectance spectrophotometer SpectroShade (LUA005, MHT Optic Research AG, Zurich, Switzerland; software version, 2.20)
			End point: end of active treatment			
Eliades 2001 [23]	Prospective split-mouth design	Group I:15	No-mix adhesive resin (Unite, 3 M, Monrovia, CA, USA) chemically cured (GC Fuji Ortho, GC Corp., Tokyo, Japan)	The resultant color differences (DE) between the interval groups were calculated	The highest differences were recorded for the baseline-debonding interval for both adhesives used. No difference was found with respect to E between adhesive materials.	Artificial accelerated photo-ageing
		Group II:15				
Trakyali 2009 [26]	RCT	Group 1:15	Transbond XT Light cure adhesive 3 M Unitek,	The resultant color differences (DE) between the interval groups were calculated	Color changes of orthodontic bonding systems induced by photoageing cannot be clinically observed.	Artificial accelerated photoageing
		Group 2:15				
		Group 3:15	Monrovia, CA, USA			
		Group 4:15	Eagle Bond American Orthodontics, Sheboygan, Wisconsin, USA			
		Group 5:15				
					Polishing with Stainbuster eliminates enamel surface roughness, which may improve light reflection.	
			Blugloo Ormco, Scafati, Italy			
			Light bond Reliance Orthodontic Products Inc., Itasca, Illinois, USA			
			Unite 3 M Unitek			
Boncuk 2014 [22]	RCT	Of the 175 teeth, 25 served as control specimens.	Group 1 (control). untreated	CIE (Commission Internationale de l’Eclairage) L*a*b* color system	Orthodontic treatment alters the original color of enamel, and both the adhesive system and the resin-removal methods are responsible for this change. When brackets are bonded with the etch-and-rinse system or the SEP, cleaning the adhesive residuals with Stainbuster burs is recommended for minimal change.	handheld spectrophotometer (SpectroShade
			Group 2. Enamel was etched with 37% orthophosphoric acid ,Transbond XT Adhesive Primer (3 M Unitek, Monrovia, CA, USA)			
			Group 3. A self-etch adhesive system (Transbond Self-Etching Primer [SEP]; 3 M Unitek) was used in conjunction with Transbond XT Adhesive Resin as with group 2.			
			Group 4.			
			20% polyacrylic acid, the brackets were bonded with light-cured resin-modified glass ionomer cement (RMGIC; Fuji Ortho LC; GC Corp, Tokyo, Japan).			
		The remaining were randomly assigned to three experimental groups (n = 150 each) with respect to the adhesive tested				Micro;
						MHT, Verona, Italy
Joo 2011 [24]	RCT	Teeth specimens were randomly divided into 9 groups of 15 teeth	Group 1: Control	CIE (Commission Internationale de	The self-etching primers system would show less stain	reflection spectrophotometer (CM- 3500d, Minolta, Osaka, Japan)
			Group 2: Transbond-F (3 M Unitek)			
			Group 3: Transbond-FP (3 M Unitek)	l’Eclairage) L*a*b* color system	susceptibility if the thin residual adhesive resin layer after debonding is removed by polishing	
			Group 4: Ortho Solo-F (Ormco Corp, Glendora, CA)			
			Group 5: Ortho Solo-FP (Ormco Corp, Glendora, CA)			
			Group 6: Transbond Plus-F (3 M Unitek)			
			Group 7: Transbond Plus-FP (3 M Unitek)			
			Group 8: Prompt L-Pop-F (3 M ESPE, Seefeld, Germany)			
			Group 9: Prompt L-Pop-FP (3 M ESPE, Seefeld, Germany)			

### Risk of bias

**Figure 2:**
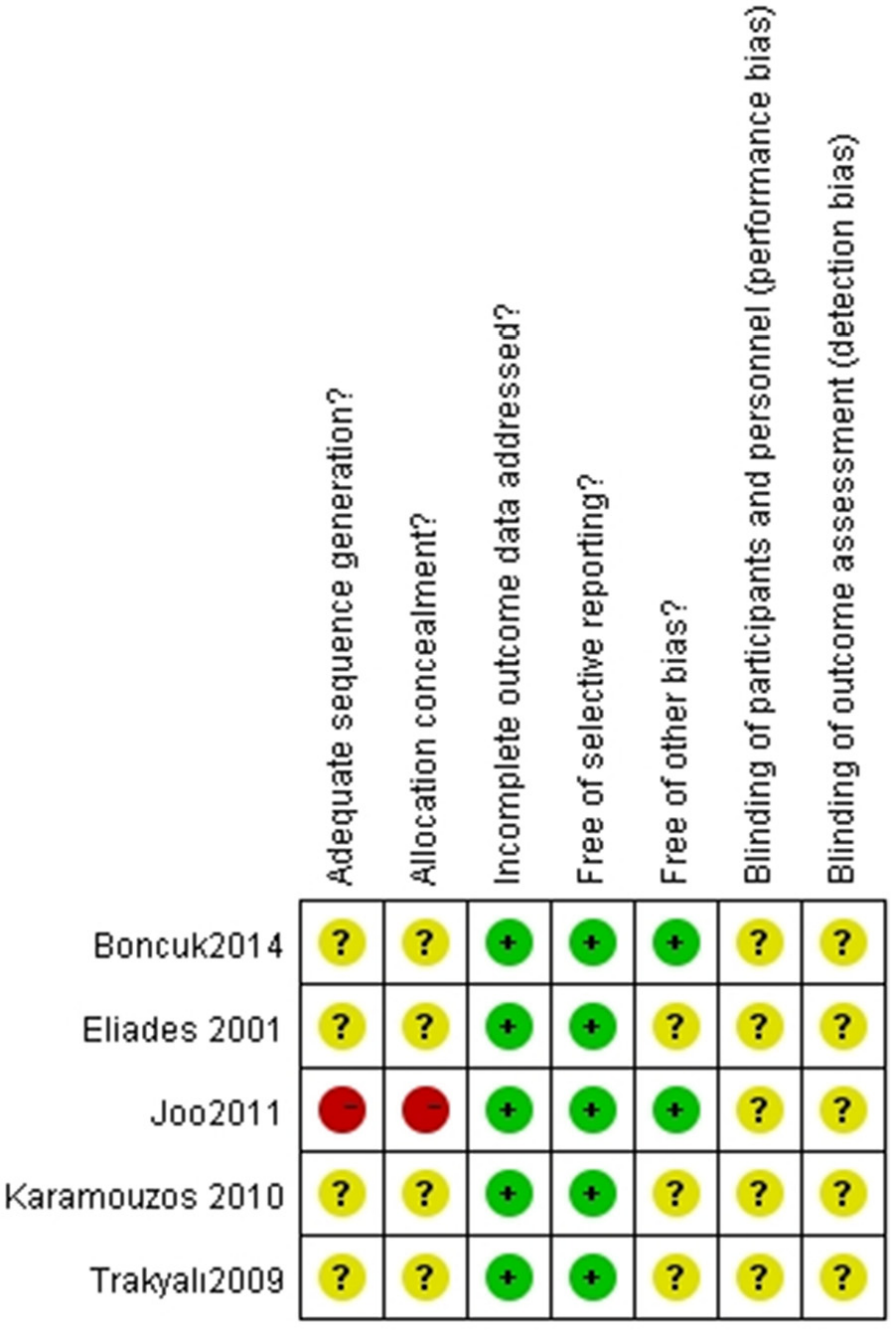
**Risk of bias summary.**

A summary of the risks of bias in the included studies is shown in Figure 2. Four of the 5 trials included in this review had at least two domains assessed as being of unclear risk of bias, and 1 of the 5 studies was assessed as being at overall high risk of bias. Two studies were prospective, and 3 were randomized. Withdrawals (dropouts) were not declared in any of the 5 studies. The most recurrent shortcomings were random sequence generation, and allocation concealment with no methods of sequence generation described. Furthermore, only 1 study declared any power analysis.

### Description of outcomes

The effects of orthodontic adhesives, and the effects of resin removal techniques on enamel color alteration were evaluated.

#### Orthodontic adhesives

Four of the 5 studies investigated orthodontic adhesives [22,23,25,26]. Boncuk et al. [22] compared an etch-and-rinse adhesive system, a self-etch adhesive system (SEP), and a resin-modified glass ionomer cement. They reported that orthodontic treatments altered the original color of enamel, and that both the adhesive systems and the resin-based method they investigated were responsible for changes. Eliades et al. [23] evaluated the enamel color changes associated with bonding of brackets with a “No-mix” adhesive resin (Unite) and a glass-ionomer adhesive (GC, Fuji Ortho). They found that all differences noted exceeded the threshold for clinical detection. The greatest differences were recorded for the baseline-debonding interval, for both the adhesives investigated.

Karamouzos et al. [25] found that chemically cured resin was associated with greater color changes than light-cured composite. Trakyali et al. [26] compared five different adhesives (Transbond XT, Eagle Bond, Light Bond, Blugloo, Unite), and found that ΔE values between the first and second measurements increased in the Transbond XT, Eagle Bond, and Light Bond groups. The highest mean ΔE value was 1.51 ± 1.15, in the Transbond XT group. No clinically significant ΔE values were observed. They concluded that color changes associated with orthodontic bonding systems induced by photoageing cannot be clinically observed.

#### Resin removal techniques on enamel color alteration

Three of the 5 studies investigated resin removal techniques [22,24,26]. Trakyali et al. [26] reported that polishing with “Stainbuster” eliminates enamel surface roughness, which may improve light reflection. Boncuk et al. [22] reported that when brackets are bonded with the etch-and-rinse system or the SEP, cleaning the adhesive residuals with Stainbuster burs is recommended, to minimize change. RMGIC can be safely cleaned with tungsten carbide burs. Joo et al. [24] reported that the SEP was associated with less stain susceptibility if the thin residual adhesive resin layer remaining after debonding was removed by polishing.

## Discussion

The relationship between tooth color changes and orthodontic treatment with fixed appliances remains controversial. Some investigators have concluded that bonding and debonding procedures alone did not seem to have a significant influence on the color of human tooth enamel. Conversely, other studies have shown that enamel color variables were significantly affected by these procedures. This systematic review is the first to evaluate color alterations in enamel following the use of different orthodontic bonding resins and different clean-up procedures, and it revealed some interesting findings.

### Quality of the evidence

The methodological limitations were extensive, and this influenced the quality of the evidence. Four of the 5 trials included in this review were assessed as being associated with an unclear risk of bias, and the remaining trial was deemed to be associated with a high risk of bias. A very serious limitation of most studies was the lack of sufficient information in the trial report to enable reviewers to determine the risk of bias. Intention-to-treat analysis would be a more appropriate technique, ensuring consideration of all subjects initially randomized, and maintaining the benefits of randomization throughout the trial.

Further prospective research in this area should be reported in accordance with the CONSORT guidelines. This would improve the quality of research studies, permitting further meta-analyses, and render components of the research including methods of randomization and allocation concealment more transparent. Many of the trials were small, and may have had an insufficient number of participants to determine a statistically significant difference between interventions, or between interventions and control conditions, if in fact this was present (type 2 error). Sample size calculations were only reported in 1 of the 5 trial reports included in this review.

### Summary of main results

In reviewing the studies, and with the limitations listed above taken into consideration, the available evidence seems to support the fact that orthodontic treatment alters the original color of enamel, and both the adhesive system and resin-removal methods are responsible for this change. Four of the 5 studies reviewed [22,23,25,26] investigated the effects of orthodontic adhesives on enamel color alteration. All of these indicated that adhesive was associated with changes in the CIE color parameters of natural teeth.

Comparisons of adhesive materials demonstrated that chemically cured (CC) resin exhibited greater color changes than light-cured (LC) composites. Among the LC composites, Trakyali et al. [26] assessed 5 different adhesives (Transbond XT, Eagle Bond, Light Bond, Blugloo, Unite) and found that there was a statistically significant difference between the groups bonded with Transbond, Eagle Bond, and Reliance. This difference was due to an increase in ΔE values between the first and second measurement. Eliades et al. [23] further concluded that debonding cleaning processes involving adhesive grinding may be more invasive relative to adhesive without grinding, with regard to enamel color alteration.

However, enamel color was affected by several factors. External coloring occurs as a result of superficial absorption of food pigments, while internal coloring occurs during aging. Since most of the included studies were in vitro, the conclusions should be interpreted with caution. It must be noted that long-term resin discoloration due to absorption of colorants from the oral environment cannot be estimated. As explained previously [27-29], the lack of saliva, food coloring, and the inability to simulate the mechanical abrasion caused by brushing are limitations of this methodology. In order to gain more reliable results, more in vivo investigations (of which only one was included in this study) must be undertaken in future. Thus, the evidence to support the relationship between orthodontic adhesives and enamel color alteration is moderate. It is recommended that further well-designed clinical trials should be conducted to investigate this question.

Three studies [22,24,26] investigating the effects of resin removal techniques on enamel color alteration were identified. There is moderate unreliable evidence, based on a small study and an unclear risk of bias, that the SEP is associated with less stain susceptibility if the thin residual adhesive resin layer remaining after debonding is removed by Stainbuster, rather than tungsten carbide. This finding can be explained by the fact that Stainbuster burs yield a smoother enamel surface, and increase light reflection.

Since the sensitivity of the human eye is limited with regard to detecting small color differences, and the interpretation of visual color comparisons is subjective, color measuring instruments are used to obtain reproducible results. Color measurement was performed according to the CIELAB color scale relative to the CIE standard illuminant D65 on a reflection spectrophotometer in all included studies. A color change above the threshold (*i.e.*, ΔE = 3.7) observed in 56% of the control specimens confirms the efficacy of this method. The lack of saliva, food coloring, and the inability to simulate the mechanical abrasion caused by brushing are limitations of this methodology. In additional, it should be mentioned that these are experimental results, not clinical results. These findings emphasize the potential risk of tooth color alteration associated with fixed orthodontic treatment.

### Overall completeness and applicability of evidence

Enamel discoloration after orthodontic treatment is often overlooked in daily practice. There has been a great deal of research into developing orthodontic adhesive materials with properties that could alter the original color of enamel. While the risk of bias of all the included studies ranges from unclear to high, all of the trials had at least two uncontrolled variables, which was likely to be a confounding factor. Well-designed, randomized, controlled trials, with assessors blinded with regard to outcome measurements, and with adequate sample sizes are required, to minimize potential biases in future studies.

## Conclusions

1. This review indicated that adhesive systems and resin-removal methods may be associated with enamel discoloration, but the evidence for this was not strong.
2. The limitations of the studies included indicate that further well-designed and conducted RCTs are required, and the standardization of methodology is recommended for future studies, to facilitate comparisons of results across trials.
